# Use of health outcome and health service utilization indicators as an outcome of access to medicines in Brazil: perspectives from a literature review

**DOI:** 10.1186/s40985-019-0115-1

**Published:** 2019-12-09

**Authors:** Luisa Arueira Chaves, Danielle Maria de Souza Serio dos Santos, Monica Rodrigues Campos, Vera Lucia Luiza

**Affiliations:** 1 Federal University of Rio de Janeiro, Macaé, Brazil; 2 National School of Public Health, Oswaldo Cruz Foundation, Rio de Janeiro, Brazil; 3 Carl von Ossietzky Universität, Oldenburg, Germany; 4 Department of Social Sciences, National School of Public Health Sergio Arouca, Oswaldo Cruz Foundation, Rio de Janeiro, Brazil; 5 Department of Medicines and Pharmaceutical Policies, National School of Public Health Sergio Arouca, Oswaldo Cruz Foundation, Rio de Janeiro, Brazil

**Keywords:** Indicators, Access to medicines, Evaluation, Literature review

## Abstract

**Background:**

To guarantee the right to health, the health system must also ensure access to medicines. Several financial arrangements to provide these technologies are implemented and range from the direct (either total or partial) to indirect payment by the patient, being necessary to evaluate its effect on access to medicines. However, to ensure access to medicines is not just about ensuring its availability, as this only materializes in its use. Thus, evaluation studies of interventions in access to medicines have been using indicators related to the health results and use of health services as its outcomes. Furthermore, as this relationship is not direct, it is important to critically assess the adequacy of these tools to measure this phenomenon and, additionally, the ability to use it in the Brazilian scenario. Therefore, this study sought to identify, describe, and analyze the use of these indicators as medicine access outcomes, through a review of the scientific literature.

**Methods:**

An extensive literature review was done using a bibliographic database for a systematic review. The references were selected based on inclusion and exclusion criteria, and the indicators from the papers retained were analyzed using the parameters of validity, measurability, reliability, and relevance.

**Results:**

We have analyzed over 12,000 references of which 30 references were included, describing the use of 49 health outcomes and health service use indicators. The majority reported the use of health service utilization measures. In our evaluation, the best indicators for assessing the effects of co-payment intervention on access are the ones aimed at specific populations or symptomatic health conditions in which the response to the therapeutic treatment is known and occurs in a short period of time. It was evident the lack of information on the indicators analyzed as well as the limitation of the Brazilian secondary databases for its calculation.

**Conclusions:**

This research showed the variety and heterogeneity of the indicators used in scientific studies. The best indicators for access to medicines are sought to measure the use of health services for symptomatic health conditions that are quickly responsive to pharmacological treatment, while the indicators related to worker productivity loss was the most suitable for health outcomes.

**Electronic supplementary material:**

The online version of this article (10.1186/s40985-019-0115-1) contains supplementary material, which is available to authorized users.

## Background

Access to medicines is fundamental to guarantee the right to health [1], since these are necessary technologies for the treatment and/or cure of several diseases. As a concept, it can be seen as the relationship between the supply and the need for these products, as long as it is dispensed with the proper information and quality [2]. However, availability on the shelves is insufficient to guarantee access to medicines, because in our understanding, the provision of medicines means much more than just giving the product to people. We advocate for the importance of access to medicines in order to provide better quality of health to people. Therefore, access to medicines is only achieved if medicines are actually used.

The supply of medicines can occur directly by the public service, privately, or a mix of the two previous forms; the latter is currently the case of Brazil [3]. Regarding the form of payment, it can be direct (integral or partial), made at the point of provision and linked to specific products; or indirectly, through the payment of taxes or private insurance that will subsidize the purchase of medicines, which, then, are generally available without any payment from the citizen at the point of access. This is called co-payment, when users partially participate in the acquisition of medicines at the dispensing points [4].

Co-payment is a cost-containment strategy for the third-party payer (either the government or an insurer), but it is also considered an access strategy because it can be a sustainable financing mechanism, as well as promoting the rational use of medicines, since it would inhibit the unnecessary use of medicines by placing a financial barrier on the patient [5]. In fact, over the years, several co-payment strategies have been proposed and implemented in health systems [4, 5].

The types of co-payment can be presented as follows [4]:

- Cap: The third payer pays up to a maximum limit, either financial or number of units. After the limit is reached, the individual assumes the total cost of the drug expenses;
- Ceiling: The individual or his/her family pays up to a financial limit, and once this limit is reached, the third party starts to cover the costs. This form intends to protect the individual and his/her family from catastrophic expenditures;
- Fixed co-payment: The individual pays a fixed amount per prescription or per medicine(s);
- Coinsurance: The individual pays a percentage of the total cost of his/her treatment;
- Tiered co-payment: Medicines are classified in different value ranges. This type of cost sharing often intends to influence the purchase and prescription of generic versions or those of high therapeutic value, classifying them at the lowest price values, while it places the reference medicine and/or the ones considered as low therapeutic value in the upper bands.

Understanding the effects of these widely used co-payment modalities on access to medicines is fundamental in the discussion of guaranteeing the right to health. One way of evaluating its effects is through the use of indicators. Indicators are tools that aim to express an effect of the phenomenon evaluated [6]. Some studies have already pointed out the diversity and heterogeneity in the application of drug use indicators in the scientific literature [7–9]. These studies demonstrate that the critical evaluation of the published articles becomes a fundamental part for the construction of new research, in order to highlight differences and consensus in the literature, showing possible methodological paths to follow.

In addition, given that access to medicines is the evaluated effect, it is important to consider these tools in its practical use on research, data source needed, and beyond its dimensions of availability or price. If this phenomenon is understood as one component to achieve better health for all, it must be reflected in indicators that express how it influences the health condition of the population served.

Thus, this research proposes to critically analyze the indicators used for health outcome and health services utilization in evaluation studies of co-payment strategies for medicines and discuss whether it is suitable to measure access to medicines in Brazil.

## Methods

This was a literature review study based on the references of a bibliographic database generated to update the systematic review “Pharmaceutical Policies: Effects of Cap and Co-payment on Rational Drug Use” [10], here referred as the source study. This bibliographic database was chosen because it is a comprehensive and updated literature search on the subject until 2013. In March, 2018, the database was updated using the same search strategy as the source study to include bibliographies published until 2017. Unfortunately, it was not possible to update all the bibliographic databases because it changed the search tool, becoming impossible to replicate the previous search strategy, or because of lack of access.

### Data source and inclusion/exclusion criteria

We analyzed 268 papers/documents from the whole bibliographic database from the source study, which consists of 8381 documents recovered from the following scientific and gray literature sources without distinction of country or language, published until 2013: Cochrane Library, MEDLINE In-Process & Other Non-Indexed Citations (Ovid), MEDLINE 1946 (Ovid), Embase (Ovid), International Political Science Abstracts (IPSA) (Ebsco), EconLit 1969 (ProQuest), Worldwide Political Science Abstracts (ProQuest), PAIS International, Public Affairs Information Service (ProQuest), INRUD Bibliography, International Network for Rational Use of Drugs, WHOLIS (VHL), LILACS (Latin American and Caribbean Health Sciences Literature), AIM (AFRO) (Global Health Library WHO), IMEMR (EMRO) (Global Health Library WHO), IMSEAR (SEARO) (Global Health Library WHO), WPRIM (WPRO) (Global Health Library WHO), PubMed (relevant works not indexed in MEDLINE), SCOPUS, OpenGrey, Jolis, SciELO (BIREME), ISI Web of Knowledge, OECD Library, OECD, WHO, World Bank e-Library, and World Bank Documents & Reports. The search keys are described in the source study [10].

The following bibliographic databases were searched during March, 2018: Cochrane Library, MEDLINE In-Process & Other Non-Indexed Citations (Ovid), MEDLINE 1946 (Ovid), INRUD Bibliography, International Network for Rational Use of Drugs, WHOLIS (VHL), LILACS (Latin American and Caribbean Health Sciences Literature), AIM (AFRO) (Global Health Library WHO), IMEMR (EMRO) (Global Health Library WHO), IMSEAR (SEARO) (Global Health Library WHO), WPRIM (WPRO) (Global Health Library WHO), PubMed (relevant works not indexed in MEDLINE), SCOPUS, OpenGrey, Jolis, SciELO (BIREME), ISI Web of Knowledge, and World Bank e-Library. This update resulted in 6826 references that after cleaning for duplicates and excluding for 2018 publications, added 4165 references to the bibliographic database. These updated documents were screened according to the source study protocol as explained in the next paragraph.

The first screening was based on Cochrane criteria for systematic reviews [11], and the application of the inclusion and exclusion criteria was done by pairs of authors, who worked independently and blindly. After the independent evaluation of each author, the results were compared and the divergences resolved by consensus. If consensus was not reached among peers, the lead author of the source study was called upon to collaborate in the final decision.

In the first screening, the title and the abstract were analyzed according to the following: Purpose: include/change the co-payment mechanism directly affecting medicines and/or increase its value, either for the patient or the provider;

- Outcomes: Outcome measures had to be objective and related to results in health, use of health services, use of medicines, and/or cost;
- Study population: Only regional or national studies were included. Restricted populations were included if it was a pilot study for further implementation in a broader population;

The second screening was carried out specifically for the objective of this study. This selection was done based on the full text and reviewed by one of the co-authors according to these inclusion criteria:

- Studies to which we had access to the full text;
- Studies that used indicators of health outcomes and/or use of health services as an outcome;

Also, we only considered quantitative studies.

Congress presentations, research reports, and review studies were excluded.

### Data extraction

Data from the selected publications were extracted in three worksheets related to the characteristics of the articles and the indicators used in the selected studies. The first worksheet was related to the general data of the study and the other two to the specific data about the indicators: one for indicators related to health outcomes and one for health service utilization.

Regarding the characteristics of the studies, information was extracted on nationality of the first author, place of study, description of the drug access mechanism before and after the intervention, implementation per year of the intervention, type of co-payment assessed, study design, outcomes, and comments. The co-payment mechanism was classified as cap, ceiling, fixed, coinsurance, tiered co-payment, or a mix of those types. Regarding the type of study, the classification of the Cochrane Handbook for Systematic Reviews of Interventions was used [11].

Regarding the information about indicators, the following data were extracted based on the information stated on the paper: name (literal translation of the original name), definition, calculation formula, source of data, scale or hypothesis for interpretation of its results (when applicable), if the indicator was validated or not, bibliographic reference linked to the indicator, and, finally, any comments considered relevant to the analysis.

### Analysis

The analysis aimed to discuss the applicability of the indicators in Brazil as an outcome of access to medicines based on the information extracted. Additionally, when the paper linked a reference to the indicator used, we retrieved it as well. It was used as parameters for the characteristics of validity, reliability, relevance, and measurability of the indicators.

In this study, we used the definition of the attributes of the health indicators of the Inter-agency Network of Information for Health (RIPSA) [6], in which:

- Validity is the ability to measure what is intended. Two criteria can be used for this attribute, specificity, and sensitivity. Specificity refers to the ability to measure only the desired phenomenon, while sensitivity refers to the ability to measure the desired phenomenon;
- Reliability refers to the degree of comparability, which means that when used in similar situations its results should be similar;
- Measurability refers to the availability of the sources necessary for its measurement. In the case of the present study, this analysis was based on the existing databases in Brazil;
- Relevance refers to the estimated importance of the indicator regarding the priorities of the health system.

In addition, the quality of each attribute was classified according to the criteria, heuristically defined as good, intermediate, and low, followed by a brief explanation of its attribution. For the qualification of the attribute of validity, it was considered how much that indicator could be used as an outcome measure of access to medicines. In order to assess the reliability attribute, it was considered how much the result of that indicator would remain stable, both in the case of its measurement in different regions in Brazil and in the comparison between countries, considering the differences in context. For the measurability attribute, the availability of data required for the calculation of the indicator was evaluated in the Brazilian scenario, that is, to what extent the calculation is operationally feasible in the country. Finally, the evaluation of the relevance of the indicator was based in the importance it would have for the analysis of access to medicines in Brazil. This country was chosen because it is the residence country of coauthors of this paper and all have expertise in the utilization of the Brazilian information system for research, giving them a great base for discussing it.

The indicators that had the same reported name were grouped together, regardless if they had differences in their reported definition or calculation formula. We also have classified the health service use indicators into categories referring to the type of service they measured: hospitalization, outpatient services, emergency, total health services (outpatient + emergency + hospitalization), hospital services (emergency + hospitalization), laboratory and diagnostic services, and home visits. This grouping was done because each one responds differently to access to medicines levels.

## Results

**Fig. 1:**
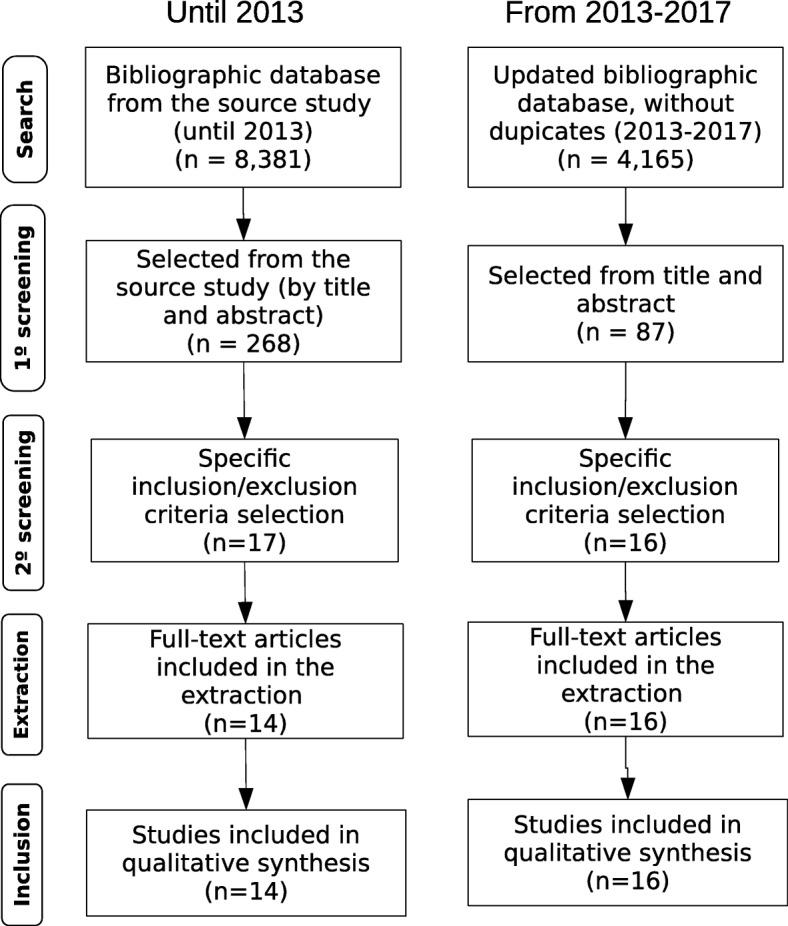
Study flow of the paper selection process

Figure 1 shows the selection result of the application of the inclusion/exclusion criteria in the analyzed references. The main reasons for exclusion were, consecutively, not using health outcomes and/or health services utilization as study outcomes, the interventions analyzed were not related to co-payment for medicines, and, finally, were not intervention studies.

A detailed description of the characteristics of included studies is presented in Additional file 1. The majority of studies (29) retained were conducted in high-income countries, mainly the USA (20) and Canada (5). Regarding the study design, all of them used observational approaches to evaluate the interventions. The studies mainly used before and after designs (18).

Among the 30 included references, we extracted data of 15 health outcomes and 34 health service utilization indicators. The latter was classified into seven categories, presented in Table 1.

**Table 1:** Health service utilization indicators per category

Categories	Number of indicators
Hospitalization	11
Outpatient services	12
Emergency	6
Home visits	2
Total health services (outpatient + emergency + hospitalization)	1
Hospital services (emergency + hospitalization)	1
Laboratory and diagnostic services	1
Total	34

The most frequent indicators were related to evaluating the use of hospitalization services, emergency use, and outpatient consultations as outcomes of the co-payment changes. Only one study [12] linked a bibliographic reference to the proposed indicator. All of the indicators are described in Table 2.

**Table 2:** Description of health services utilization indicators

Name	Definition	Calculation	Source of Data	Interpretation/ Scale
Emergency				
Use of emergency services [13]	Dichotomy	Count: dichotomy	CMS Medicaid Analytical Extract database	NI
Number of visits to the emergency department related to the DM [14]	Visits related to diabetes when ICD was the primary, second or third diagnosis	NI	NI	NI
Change in the annual number of emergency care [15]	NI	NI	Administrative database	Hypothesis: the copayment increase will not increase the use of medical and non-pharmaceutical services
Number of visits to the emergency department [12, 16–18]	Frequency of visits to the emergency department in the year after discharge [16]	NI	Administrative database [16]	NI
	NI [17]		Medical Expenditure Panel Survey (MEPS)^1^ [17, 18]	
	Annual number of visits [18]		NI [12]	
	Treat and release only [12]			
Proportion of visits to the emergency [19]	NI. It is not clear, however the name leads to a presumption that it is the proportion of the studied patients that had a visit to the emergency	NI	Administrative base of individual data linked to the registry of cancer from 1999 to 2004 of Georgia, South Caroline and Texas	Hypothesis: although the treatments directly related to cancer are exempt of copayment, the patients need other medicines that are subject to cost sharing.
Emergency admission [20]	Emergency hospital admission for any reason	Emergency hospital admissions/1000 patient-year	PharmaNet database	NI
Hospitalization				
Hospitalization [14, 20, 21]	Visits related to DM when ICD was the primary, second or third diagnosis [14]	NI	NI [14]	NI
	Emergency hospitalization when the primary reason was a chronic and obstructive pulmonary disease bronchitis, asthma or emphysema [20]		PharmaNet database [20]	
	Mean number of visits [21]		U.S. Renal Data System (USRDS)^2^ [21]	
Number of hospitalizations [17, 18]	Annual number of visits. The number of discharges included those hospitalizations for which the admission and discharge date were the same [18]	NI	The Medical Expenditure Panel Survey (MEPS)^1^	NI
	NI [17]			
Number of days of hospitalization [22] /Days of hospital stay [21]	NI	NI	National registry of psychoses [22]	NI
			U.S. Renal Data System (USRDS)^2^ [21]	
Changes in the annual number of hospitalization [15]	NI	NI	Administrative database	Hypothesis: the copayment increase will not increase the use of medical and non-pharmaceutical services
Hospitalization use rates [23]	Hospitalization whose diagnose code is related to depression	Monthly calculation per 1000 elderly	PharmaNet database	Unexpected consequences of the intervention, cushioning the economy with medicines
Hospital utilization [24, 25]	Demonstrate if the person was hospitalized within a month [24]	NI	Insurance companies database [24]	Unexpected consequences of the intervention, cushioning the economy with medicines [24]
	Whether the individual spent any days in the hospital during the year (probability of hospitalization) [25]		Administrative database [25]	“An offset effect could be hypothesized to exist for elderly patients in the form of reduced hospital utilization when they become eligible for high cost sharing exemption. This offset effect may arise from increased initiation of chronic treatment or improved patient compliance for effective prescription medicines under free care” [25]
Hospital admission [13, 26]	Dichotomous [13]	NI [13]	CMS Medicaid Analytical Extract database [13]	NI
	NI [26], but by the calculation formula it is clear that it is not a dichotomous indicator as defined in the other included study.	annual incidence of hospitalizations (asthma and non respiratory diseases) per 100,000 people by dividing the number of cases of disease by the midyear population estimates, and multiplying the quotient by 100,000. [26]	DATASUS^3^ [26]	
Psychiatric admission [22]	NI	NI	National registry of psychoses	NI
Risk of psychiatric admission [22]	NI	NI	National registry of psychoses	NI
Incidence of readmission for complications related to acute myocardial infarction and death [16]	Categorized at 30 days, 6 months and 1 year after discharge	NI	Discharge database	NI
Percentage of people with an inpatient admission to a hospital in 2007–09 [12]	NI	NI	NI	NI
Outpatient services				
Use of outpatient services [13, 24, 27]	Sum of outpatient monthly visits, according to the selected ICD [13]	NI	CMS Medicaid Analytical Extract database [13]	NI
	Number of use of ambulatory appointments/person/year [27]		Ambulatory services dunning data [27]	NI [27]
	Number of doctor’s appointment in an ambulatory or clinic within one month [24]		Insurance companies database [24]	Unexpected consequences of the intervention, cushioning the economy with medicines [24]
Outpatient visits [14, 21, 22]	Visits related to DM when ICD was the primary, second or third diagnosis [14]	I	NI [14]	NI [14]
	NI [22]		National registry of psychoses [22]	The intervention can create a financial obstacle resulting in an increase of the use of health services [22]
	Mean number of visits [21]		U.S. Renal Data System (USRDS)^2^ [21]	NI [21]
Number of outpatient visits [18, 21, 28]	Annual number of visits [18, 21]	NI	Medical Expenditure Panel Survey (MEPS)^1^ [18, 21]	NI
	NI [28]		National Sample Cohort^4^ [28]	
Number of visits to a physician [20]	Number of visits to a doctor	Number of outpatient visits to a doctor/1000 patient-year	PharmaNet database	NI
Number of visits to a doctor [29]	NI	NI	NI	NI
Number of physician office visits [17]	NI	NI	Medical Expenditure Panel Survey (MEPS)^1^	NI
Outpatient medical visits [16]	Defined as the frequency of outpatient medical visits in the first year after discharge. Includes visits to family doctors, interns and cardiologists in ambulatories, clinics and health centers.	NI	Administrative database	Hypothesis: the frequency of the visits should increase as a response to the pharmaceutical coverage.
Use of ambulatory healthcare services [30]	NI	NI	NI	NA
Change in the annual number of ambulatory visits [15]	NI	NI	Administrative database	Hypothesis: the copayment increase will not increase the use of medical and non-pharmaceutical services
Rate of use of clinical services [23]	Appointments with a diagnosis code related to depression	Monthly calculation/1000 elderly	PharmaNet database	Unexpected consequences of the intervention, cushioning the economy with medicines
Utilization rate of the psychiatric services [23]	Appointments with a diagnosis code related to depression	Monthly calculation/1000 elderly	PharmaNet database	Unexpected consequences of the intervention, cushioning the economy with medicines
Proportion of general or tertiary hospital utilization [28]	The proportion of general or tertiary hospital utilization among total healthcare utilization.	(the number of outpatient visits into general or tertiary hospitals per person–month/the number of outpatient visits into total healthcare utilization per person–month) × 100	National Sample Cohort^4^	NI
Total health services				
Number of use of health services/100 members/month [31]	Ambulatory appointments included, use of emergency services and hospitalization	NI	Administrative data from Oregon’s Medicaid Program	NI
Hospital Services				
Use of hospital health services [30]	Use of emergency services and hospitalization	NI	NI	NA
Diagnosis and Laboratory services				
Use of laboratory and diagnosis services [14]	Visits related to DM when ICD was the primary, second or third diagnosis.	NI	NI	NI
Home visits				
Change in the annual number of home visits [15]	NI	NI	Administrative database	Hypothesis: the copayment increase will not increase the use of medical and non-pharmaceutical services
Other visits [21]	Mean number of visits. Includes home health agency, skilled nursing facility, or hospice	NI	U.S. Renal Data System (USRDS)^2^	NI

*Subtitles: NI* Not Informed, *DM Diabetes Mellitus*, *ICD* International Classification of Diseases

^1^Annual estimates of health care use, cost, payment sources, health insurance coverage, health status, and sociodemographic characteristics for the US civilian, noninstitutionalized population [18]

^2^A national registry of subjects with end-stage renal disease based on Medicare claims. This database includes Medicare enrollment history, death dates and causes, and Medicare Parts A and B claims [21]

^3^A national database that contains information on epidemiology and morbidity of various diseases that impact on the health of the Brazilian population [26]

^4^Data, including all medical claims, from 2010 to 2013 released by the National Health Insurance Service (NHIS), which consists of details of patient healthcare utilization [28]

In the analysis of the attributes of the indicators (presented in details in Additional file 2), those classified as indicators of “emergency use” [12–19] presented good evaluations of reliability and relevance attributes, while attributes of validity and measurability were evaluated as intermediate and low, respectively.

In contrast, those categorized as indicators that measured “hospitalization” [12–18, 20–27], especially those that measured this outcome for specific causes, were evaluated as good indicators (validity, reliability, and relevance), but with low measurability due to the impossibility of identifying specific populations in databases, or the low quality of secondary databases. When the indicators in this category referred to hospitalization for all causes, measurability was assessed as high, but, as they are very general measures, their relevance and validity were low. The same occurred with the category “total health services” [31], which measured hospitalization, emergency, and outpatient services in the same indicator.

The indicators categorized as “home visits” [15, 21], “laboratory services and diagnostics” [14], and hospital services [30] had its validity attribute evaluated as low to intermediate, mainly because they were services that were not responsive to changes in medicines co-payment.

Finally, the indicators categorized as “outpatient services” [13–18, 20–24, 27–30] had a more diversified evaluation. In terms of measurability, when the indicator measured the use of services for all types of causes and for the entire population, this attribute was well evaluated; but its validity and relevance did not have good evaluations. In cases where the indicator considered only the use of these services for specific causes and/or populations, both validity and relevance were evaluated as good or intermediate, depending only on the health condition considered. However, similarly to the case of the “hospitalization” category, it presents difficulties for its use in the Brazilian scenario.

In general, it is important to highlight the lack of information in the indicators analyzed, either in its definition or even in its calculation formula.

Eight out of 34 health service utilization indicators had their formula presented. In the case of health outcomes, the scenario is not different; of the 15 indicators analyzed, only six presented the calculation formula.

Table 3 describes the indicators of health outcomes. The detailed evaluation of each one is presented in Additional file 3. Of these, three indicators used primary data collections while the others were based on secondary databases. In addition, only one of them was directly related to the use of medicines.

**Table 3:**
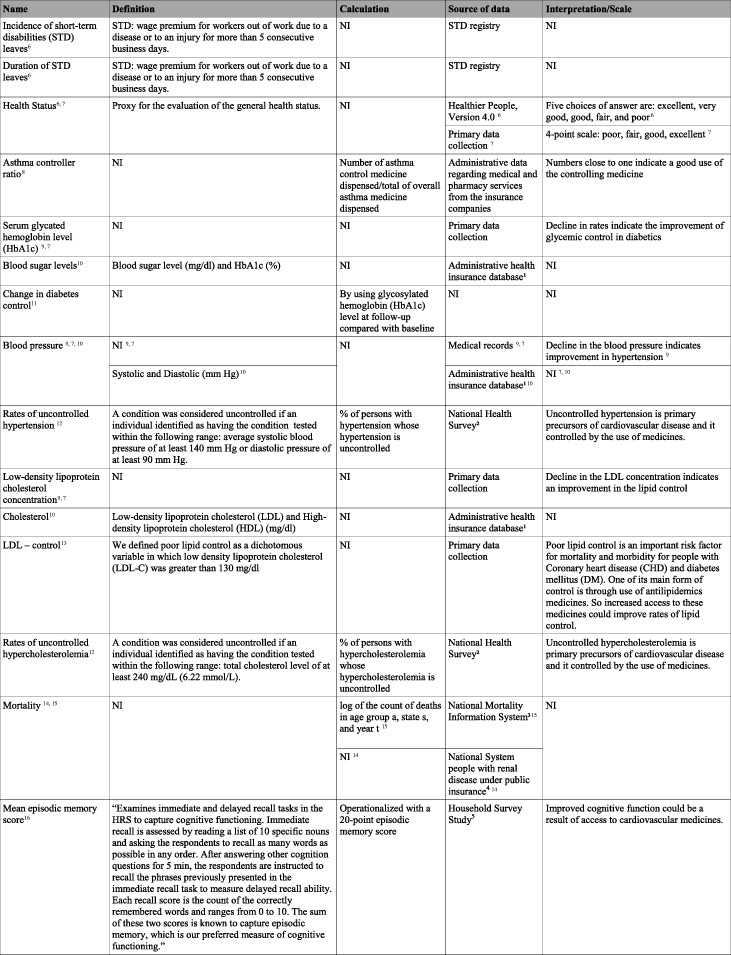
Description of health outcomes indicators

*Subtitles: NI* not informed

^1^Japan Medical Data Center (JMDC), which collects and analyzes administrative claims data on behalf of large corporate health insurers (…). The JMDC claims data cover both inpatient and outpatient spending, including prescription drug spending. The data base does not, however, contain dental claims or inpatient food and housing costs [36]

^2^National Health and Nutrition Examination Survey (NHANES) 1999–2012, a nationally representative cross-sectional survey of the noninstitutionalized civilian population [38]

^3^National Vital Statistics System of the National Center for Health Statistics for the years 2001–2008. These data provide demographic state of residence information for the universe of deaths that occurred in the USA [40]

^4^U.S. Renal Data System (USRDS) (...) is a national registry of subjects with ESRD (end-stage renal disease) based on Medicare claims (…) includes Medicare enrollment history, death dates and causes, and Medicare Parts A and B claims [21]

^5^Health and Retirement Study (HRS). The HRS is a biannual longitudinal study of 37,000 American adults aged 51 or older from 23,000 households. The study collects information on demographic and socioeconomic characteristics including health, insurance coverage, and medical care utilization [41]

^6^[21], ^7^[32], ^8^[33], ^9^[34], ^10^[35], ^11^[36], ^12^[37], ^13^[38], ^14^[39], ^15^[40], ^16^[41],

## Discussion

Despite the inclusion of bibliographical databases from low- and middle-income countries, such as Latin America and Africa, the large majority of studies included in this analysis were from high-income countries. These countries generally have comprehensive information systems, either private or governmental, containing information on the use of health services, reasons for consultation, chronic diseases, medical prescription, frequency of drug dispensing, among others. However, medium- and low-income countries commonly lack such comprehensive information systems, creating methodological obstacles in evaluation studies and limiting the capacity to conduct longitudinal studies in these scenarios. This corroborates the importance of critical evaluations of the scientific literature before the replication of these indicators in different scenarios. Our study intends to contribute to the fulfillment of this gap.

In general, health outcome indicators can be considered adequate in terms of validity and reliability, but they have limitations when considering their measurability, either because of the lack of information system in Brazil or due to the poor database quality that prevent its collection.

Burton et al. [32] took an interesting approach when using temporary disability licenses as an indicator of the effects of the change in medicines co-payment from two to three levels with increasing amounts of the patient’s share in these levels. One of the measures employed is to investigate whether there is a change in work absenteeism as a health outcome measure, based on previous findings that showed a relationship between pharmacological treatment and reduction of worker productivity loss due to illness for some diseases. The study by Burton et al. [32] did not point out any difference in the outcome in its evaluation.

Although this is a relevant indicator for evaluating health outcomes, it should be considered that, in the case of chronic diseases such as diabetes and hypertension, complications due to poor treatment adherence tend to take years to occur. Therefore, unless there is a series of data over many years, it does not seem reasonable to use these indicators in evaluations where diseases with this characteristic are the focus. The authors sought to minimize this limitation with the use of a control group.

In the case of Brazil, despite the large number of health information systems in operation, the measure of the number of “temporary incapacity leave” is not an easy task. The use of this indicator requires the linkage of several databases of different governmental institutions, becoming an obstacle for its use in the country. Likewise, if we consider the attribute of comparability between different countries, it would be important to consider the differences in labor legislation, particularly with regard to sick leaves.

Also in the aforementioned Burton et al. [32] study, the other indicator used was the worker’s perception of his/her general health status. Although not mentioned by the authors, the indicator of self-evaluation of general health status is recognized as a good approximation of the individual’s health [42, 43] and is, therefore, a good indicator, mainly regarding the attribute of validity. However, in the same way as the previous indicators, in the case of chronic diseases, in which the patient often has little or no symptoms in the initial stages even in the absence of treatment, this may not be a good indicator to evaluate access to medications. The authors seem to have obtained all the data, including this one, from electronic secondary databases. However, if it was necessary to obtain such data from primary sources, evidently it would introduce inherent methodological issues, such as increased time and cost to do so.

D’Souza et al. [34] used the asthma control ratio indicator, which expresses the proportion of medicines use in the worsening of the disease symptoms versus the use of medicines used for the chronic treatment of asthma. Therefore, values close to one would express good asthma control. In fact, a previous study showed that people with indicator values greater than or equal to 0.5 have lower probability of severe symptoms, hospitalizations and visits to emergencies, and higher scores on quality of life instruments. Thus, we can consider this a good indicator for access to medicines, presenting good attributes of validity, reliability, and relevance. However, as a weakness, especially for Brazil, it can be difficult to calculate due to the lack of a comprehensive database with individual registration of dispensing medicines for asthma (measurability). On the other hand, this indicator could easily be used in more restricted settings, such as specific health services, where manual data collection or the existence of simple databases are in place.

Finally, six studies [33, 35–39] used biologic-related measure indicators as health outcomes, such as LDL levels, glycated hemoglobin, and blood pressure. These measures are commonly used in the clinic as markers of the evolution of such diseases, quite dependent on the effectiveness of pharmacological treatment. Another known epidemiological indicator used in the included papers was mortality [21, 40]. Although the authors do not report it in the article, these indicators are known for their good attributes of validity, reliability, and relevance. However, when considering evaluation studies focusing on access to medicines, biologic measures become very difficult to use, given the absence of reliable secondary databases, especially for non-communicable diseases (NCDs**)**, as is often the case in low- and middle-income countries. Therefore, these indicators do not present a good attribute of measurability in these scenarios. The same is also valid for the cognitive function indicator used by Pak and Kim [41] in their study. Although validated and having good attributes of validity and reliability, collection of this indicator is hard in low- and middle-income settings. Mortality, on the other hand, is usually collected even in low resource settings and, in the case of Brazil, has a good and reliable database in which you can extract these data.

Other questions that arise for all health outcomes are, first, how responsive they could be to treatment adherence and, secondly, especially in the case of chronic diseases, at what time of treatment, with good adherence, it is possible to observe measurable changes in health outcome indicators. This question was not raised by any of the papers analyzed, and usually, the observed period of time takes into account other issues (often more related to the robustness of the study design and statistical analysis) than the basis of the clinical evolution of the conditions under study.

Regarding the health service utilization indicators, it is possible to say that those that considered the use of emergency services and hospitalization are the best in the expression of access to medicines. However, they are more reliable when the health condition analyzed is rapidly responsive to pharmacological treatment. That is, when in the absence of the recommended medicines, the disease causes severe symptoms that leads to the search for this type of health service, such as asthma. However, if we consider chronic health conditions, such as mild hypertension, the absence of treatment will probably lead to mild symptoms and possibly this population will not seek emergency services nor be hospitalized in the short term due to the underlying disease. Another factor that interferes with the validity of this indicator for those NCDs is the time of observation. Taking, for example, type 2 diabetes, its serious complications would take months or years to present themselves, which would require a long period of observation to capture problems in access to medications related to this health condition. In terms of measurability in the Brazilian scenario, emergency services and hospitalization indicators have as main difficulties the lack of unified information systems that register the use of emergency services, poor filling quality of the existing ones, and the possibility of identifying specific populations.

The Brazilian information system that contains data on hospitalizations, the Hospital Information System (SIH), has as its main objective monitoring, and the financial audit of the services provided aimed to financing purposes [44]. Perhaps for this reason, this database does not include the information necessary to carry out studies evaluating interventions in the health system, such as changes in access to medicines, among others. On the other hand, the SIH allows the analysis of hospitalizations for specific causes but it is not possible to identify the individual’s underlying disease (evidenced in this system by the secondary diagnosis, generally a poorly filled field), thus limiting the quality of the analysis [45]. The other limitation refers to the scope of this system, since it only presents the hospitalizations performed in the public health service or by contracted private services by the national health public system (SUS)[46]. For the measurement of the private system, it is necessary to evaluate also the database called Hospitalization Communication (CIH); however, the main limitation is the under-reporting, mainly, of estimates of hospitalization rates [46].

In the case of indicators that measure the use of outpatient services, access to medication will only be more clearly expressed if the characteristics of intervention in some way require the regular use of this service to obtain the medicines needed. For example, in the case of switching from a fixed to a tiered co-payment, in which generics are cheaper, it is possible that patients require ambulatory services to change their prescription or, if it is mandatory, the periodic validation of the prescription to obtain the medicine. Therefore, these indicators are not the best measures for the expression of access to medicines. In addition, the possibility of comparing this indicator between different countries becomes complicated, especially if there are problems in accessing outpatient services.

Another limitation for Brazil is the data source needed to calculate these measures. The main source for outpatient services in SUS is the Basic Care Information System (SIAB), which gathers information on family composition, housing and sanitation conditions, health situation, production, and composition of health teams. It is not possible to identify neither population affected by specific problems nor specific causes for the use of the service. Thus, it is only possible to gauge the number of consultations performed for the entire population, which reduces the validity and relevance of the indicator as an outcome of access to medicines. In addition to the SIAB, Brazil also has the SUS Ambulatory Information System (SIA), which aims to record outpatient visits throughout the Brazilian territory for administrative control of outpatient production [47]. However, this database has similar limitations to SIAB, despite being possible to account for specific causes from the list of procedures. The other indicators of health service use are either very generalist or use of health services that are not responsive to changes in access to medicines.

Balkrishnan et al. [30] and Hartung et al. [31] used a composite of indicators, the first combining emergency and hospitalizations services and the second combining ambulatory appointments in addition to emergency and hospitalizations services. Both composite of indicators present reliability problems, have low validity, and relevance to express access to medicines, resulting in unreliable results. The indicators that measured outpatient services and diagnosis and outpatient visits [14, 15] are not good because they do not respond to access to medicine changes. In addition, they present serious problems for the comparison of their results between different regions of the country, due to differences in the offer and access to these services across the Brazilian regions.

An important finding of our research is the poor reporting of outcome measure in the scientific literature. The studies analyzed provided little information about the calculation formula and even the indicator definition, resulting, sometimes, in not enough material to decide whether the indicators reported in different studies could be comparable. These results are consistent with previous works related to indicators of medicines use [8, 9]. Considering that indicators are synthesis measures of the outcome evaluated, their choice should take into account the attributes of the indicator and this analysis can only be fully performed when its information are evidenced in the scientific manuscript. The lack of information can lead to duplicate names of indicators and their respective calculation formulas, as evidenced by Hess et al. [8] in medicines use indicators. We also perceive as consequences the difficulty of replicating these indicators in other studies, and the generation of evidence, by comparing and compiling studies’ results, due to the multiplicity of the indicators used. In this sense, Brazil has already made efforts to standardize health nomenclatures and indicators in order to foster the management and evaluation capacity of relevant public policies [6].

Another limitation is the lack of in-depth clinical knowledge on the health conditions analyzed by the authors of this article (which could be considered as a misclassification bias). Although the evaluation of the attributes of each indicator has been discussed and reviewed with specialists in epidemiology and evaluation studies, none of them had an in-depth knowledge about the physiopathology and clinical aspects of the health conditions reported in the retained papers. Thus, there may be flaws in the evaluation of the responsiveness of the clinical condition to the pharmacological treatment, sometimes expressed in the indicators.

As it was used a bibliographic database built with the objective to review the literature of the effects of co-payment in the rational use of medicines, it is possible that some study was not retained in the search (selection bias). However, as the search that resulted in this database was quite comprehensive, it is reasonable to believe that its impact, if any, has been minimal. Variations in the health care offer were seldom addressed by the authors of the retained studies. All were from high-income countries, leading us to believe that they are based on the assumption of a stable and adequate supply for the health services demand in their contexts. Another point is that in nearly all studies retained, there was little overlap between the access to medicines’ mechanisms, and the databases used seemed quite comprehensive, making it easier to attribute the observed outcomes to the specific interventions.

We analyzed the applicability of the indicators found in scientific publications to the Brazilian context. However, we believe that this does not narrow the discussion since we approached it under a health system evaluation perspective and this case may be useful to other middle-income countries. These are likely to have information systems in place, but generally not as structured and comprehensive as in high-income countries, source of the majority of scientific publications.

Finally, when analyzing the indicators of studies retained in this research, it was possible to notice that, in general, the authors assume that adherence to treatment is related only to financial obstacles in obtaining it, disregarding the psychological aspects related to adherence to medicines treatment, which is the main limitation in the use of these indicators. On the other hand, the approach that access to medicines can be expressed in health results and use of health services includes a view that access goes beyond the simple availability of the product and therefore presents great potential for assessment of the effectiveness of medicines access policy at national and regional levels.

## Conclusion

Our findings show the variety, heterogeneity, and poor communications of the health service utilization and health outcomes indicators used in the scientific literature to assess the effects of copayment interventions in access to medicines.

Among the analyzed indicators, it is possible to say that, perhaps the most adequate to the Brazilian reality, both in terms of validity and of measurability, are those related to the use of hospital services, but only when analyzed under a specific, symptomatic, and rapidly responsive to the pharmacological treatment health condition, like asthma.

It is also important to highlight the interesting indicator of health outcomes related to loss of worker productivity. However, as well as the indicators of health service use, this will only express more clearly the access to medicines under specific conditions, such as migraine, allergic rhinitis, and asthma, as pointed out by the previous work of Burton et al. [48].

As synthesis measures, the indicators should be used carefully and in line with the study objectives, considering the local context, the intervention characteristics, and the measurement possibilities, to ensure that it can effectively express the intended outcome. To date, this is the first critical analysis in scientific research of the use of health and health service utilization indicators as an outcome of access to medicines, and therefore, we expect that these results may support new research in this theme.

## Additional files

Additional file 1: Characteristics of the evaluated intervention. This box presents detailed information about the interventions evaluated in the papers retained in this research. (PDF 83 kb)

Additional file 2: Evaluation of health services utilization indicators. This box presents the detailed evaluation made about each indicator of health services utilization outcome from the papers retained in this research. (PDF 63 kb)

Additional file 3: Evaluation of health outcomes indicators; his box presents the detailed evaluation made about each indicator of health outcome from the papers retained in this research. (PDF 63 kb)

## Data Availability

The datasets used and/or analyzed during the current study are available from the corresponding author on reasonable request.

## Abbreviations

CIH: Hospitalization Communication (*Comunicação de Internação Hospitalar*)
NCD: Non-communicable diseases
RIPSA: Inter-agency Network of Information for Health (*Rede Interagencial de Informações para a Saúde*)
SIA: Ambulatory Information System (*Sistema de Informação Ambulatorial*)
SIAB: Basic Care Information System (*Sistema de Informação da Atenção Básica*)
SIH: Hospital Information System (*Sistema de Informações Hospitalares*)
SUS: Brazilian Health National System (*Sistema Único de Saúde*)
