# Supplementary material for: Use of health outcome and health service utilization indicators as an outcome of access to medicines in Brazil: perspectives from a literature review
Source: Public Health Rev. 2019 Dec 9;40:5. doi: 10.1186/s40985-019-0115-1 (PMC6902426; doi:10.1186/s40985-019-0115-1)
Supplement: Supplementary file 1 — Characteristics of the evaluated intervention. This box presents detailed information about the interventions evaluated in the papers retained in this research. (PDF 83 kb) [file 40985_2019_115_MOESM1_ESM.pdf]

Additional file 1: Characteristics of the evaluated intervention

ID	Title	Place of study (Country)	Description of the drug access mechanism before the intervention	Intervention (description)	Year of the intervention implementati on (4 digits)	Study design
Balkrishna n2001	Effect of prescription benefit changes on medical care utilization in a Medicare HMO population	USA	1) $500 annual limit, $6 co-payment for generics and $12 for reference medicines;<br>2) $200 quarterly limit, $7 for generics and $15 for reference medicines.	1) $200 quarterly limit, $7 for generics and $15 for reference medicines;<br>2) $25 monthly limit for reference medicines, $5 co-payment for generics and $15 for reference medicines	1. 1998;<br>2. 1999	RMS
Burton2008	The association between a tiered pharmacy benefit plan and medication usage, health status, and disability absence days - One employer's experience	USA	Tier co-payment in two levels:<br>1) Between US$ 8 for 30 days filling or US$ 15 for 90 days filling;<br>2) US$ 10 for 30 days filling and US$ 40 or 90 days filling.	Tier co-payment in three levels:<br>1) US$ 10;<br>2) US$ 20-25;<br>3) US$ 35-40.	2003	CRM
Chandra20 07	Patient Cost-Sharing, Hospitalization Offsets, and the Design of Optimal Health Insurance for the Elderly	USA	$5 for generics;<br>$10 for reference medicines.	$5 for generics;<br>$15 for reference medicines in the formulary;<br>$30 for reference medicines not in the formulary<br>$1000 deductible in 2001	2001/2001	CBA

Additional file 1: Characteristics of the evaluated intervention

ID	Title	Place of study (Country)	Description of the drug access mechanism before the intervention	Intervention (description)	Year of the intervention implementation (4 digits)	Study design
Dormuth2008	Emergency hospital admissions after income-based deductibles and prescription copayments in older users of inhaled medications	Canada	Elderly (≥65 years) had full coverage for medicines	**Copay**: Low-income seniors pay $10/prescription (in the first 20 of the year) while the other seniors pay $25/prescription for the first 11 prescriptions of the year. After these limits, the prescriptions become exempt of co-payment.<br>**IBD**: Based on family income implemented with 3 components: 1) Family deductible of 0 to 2% depending on the family income; 2) 25% payment of the prescription cost after passing the amount of the deductible and; 3) Ceilling equal to 1.25, 2 or 3% of the income.	2002(Copay)<br>2003 (IBD)	CRMS
Elhayany2011	Addressing healthcare inequities in Israel by eliminating prescription drug copayments	Israel	Medicines co-payment	100% co-payment subsidy of for low-income patients with hypertension, diabetes and/or dyslepidemia.	2006	RMS
Farley2010b	Medicaid prescription cost containment and schizophrenia: A retrospective examination	USA	US$1,00 Reference medicines co-payment	a) Cap of 7 prescriptions/member/month;<br>b) Mandatory pre-authorization for monthly dispensations above 5 prescriptions;<br>c) Increase from $1 to $3 for reference medicine/dispensation;<br>d) Compulsory dispensing of generics;<br>e) 34 days restriction per dispensation;<br>f) Reduction in the pharmacies dispensing rate and physician reimbursement.	2002	CRM

Additional file 1: Characteristics of the evaluated intervention

ID	Title	Place of study (Country)	Description of the drug access mechanism before the intervention	Intervention (description)	Year of the intervention implementation (4 digits)	Study design
Hartung2008	Impact of a Medicaid copayment policy on prescription drug and health services utilization in a fee-for-service Medicaid population	USA	NI	Co-payment:<br>$2 for generics;<br>$3 for reference medicines.	2003	ITS
Johnson1997	The Effect of Increased Prescription Drug Cost-Sharing on Medical Care Utilization and Expenses of Elderly Health Maintenance Organization Members	USA	A) $1/prescription (1987), $3/ prescription (1988);<br><br>B) 50%/ dispensing with cap of $25/dispensing	A) $5/prescription;<br><br>B) 70%/ dispensing with cap of $30/dispensing	A)1988<br><br>B) 1990	CBA
Li2007	The impact of cost sharing of prescription drug expenditures on health care utilization by the elderly: Own- and cross-price elasticities	Canada	Dispensing rate until annual maximum of CAN$200	A) Maximum of CAN$ 25/dispensing and annual cap of CAN$275;<br>B) Maximum of CAN$ 10/ dispensing and annual cap of CAN$200;	2002	BA
Livingstone 2004	An investigation of the impact of supplementary health benefits for low-income families in Saskatchewan	Canada	Ceiling of $850/semester.<br>Above that, the family pay 35% of the medicines' cost.	Familiar ceiling of $100/semester.<br>Above that, the family pay 35% of the medicines' cost.	1998	BA
Nair2009b	Prescription copay reduction program for diabetic employees: Impact on medication compliance and healthcare costs and utilization	USA	a) Annual deductible of $100/individual or $300/family.<br>b) Co-payment:<br>$10 for generics;<br>$20 for selected reference medicines;<br>$30 for the other reference medicines.	All medicines and supplies needed to control diabetes at the lowest level of co-payment, $10 at pharmacies.	2005	BA

Additional file 1: Characteristics of the evaluated intervention

ID	Title	Place of study (Country)	Description of the drug access mechanism before the intervention	Intervention (description)	Year of the intervention implementation (4 digits)	Study design
Pilote2002	The effects of cost-sharing on essential drug prescriptions, utilization of medical care and outcomes after acute myocardial infarction in elderly patients	Canada	Co-pay for seniors $ 2/prescription up to $100 maximum per year. Low-income seniors and pensioners were exempt from co-payment.	A) Payment of 25% of the amount of the prescription with a ceiling of $200, $500 or $750, depending on the income. Quarterly co-payment and deductible;<br>B) Annual deductible: $0-350 depending on income. Monthly co-payment and deductible.	A)1996<br>B) 1997	BA
Subramanian2011	Impact of medicaid copayments on patients with cancer: Lessons for medicaid expansion under health reform	USA	A) Co-payment of $ 0.50 for medicines;<br>B) No co-payment for health services.	A) $ 0.75 for selected generics and reference medicines;<br>B) $ 3.00 for unselected references;<br>C) Introduction of various co-payments ($ 12.50- $ 2.00) for use of health services.	2002/2003	CBA
Wang2010b	Impact of drug cost sharing on service use and adverse clinical outcomes in elderly receiving antidepressants	Canada	Full cover. $7 payment as dispensing fee.	A) Co-pay of CAN$25 or CAN$10 for seniors and low-income population;<br><br>B) Deductible based on income. Once reached, 25% payment of the total cost. When the ceiling is reached, the insured has full coverage.	A) 2002<br>B) 2003	ITS
Li2013	Medicare part D is associated with reducing the financial burden of health care services in medicare beneficiaries with diagnosed diabetes	USA	NI	Medicare Part D (not described)	2006	ITS/ RMS
Kircher2014	Impact of Medicare Part D on out-of-pocket drug costs and medical use for patients with cancer.	USA	NI	Medicare Part D (not described)	2006	CBA/ BA

Additional file 1: Characteristics of the evaluated intervention

ID	Title	Place of study (Country)	Description of the drug access mechanism before the intervention	Intervention (description)	Year of the intervention implementati on (4 digits)	Study design
Maciejews ki2014	Value-based insurance design program in north Carolina increased medication adherence but was not cost neutral.	USA	Generics with some copay and branded name medicines in tier 3	Generics with no copayment and branded name medicines passed to tier 2 (cheaper) for medicines to treat hypertension, hyperlip-idemia, diabetes, and congestive heart failure	2008	CBA/ BA
Park2015	Health Costs and Outcomes Associated with Medicare Part D Prescription Drug Cost-Sharing in Beneficiaries on Dialysis.	USA	NI	Medicare Part-D. In 2007, the gap interval was defined as out-of-pocket spending on medicines between $799 and $3,850,6 where beneficiaries were responsible for all of their drug costs during this gap.	2007	CBA/ BA
Comaru201 6	Free asthma medications reduces hospital admissions in Brazil (Free asthma drugs reduces hospitalizations in Brazil).	Brazil	Free of charge at public institutions, costharing for some medicines at private pharmacies (90% subsidized) or out-of-pocket	Free of charge at private pharmacies for asthma medicines	2011	CBA/ BA
Fukushima 2016	Patient cost sharing and medical expenditures for the Elderly	Japan	30% coinsurance	10% coinsurance after turning 70 years old	NI	ITS/ RMS
Puig-Junoy2016	Free medicines thanks to retirement: Impact of coinsurance exemption on pharmaceutical expenditures and hospitalization offsets in a national health service	Spain	40% coinsurance rate for outpatient prescription medicines; 10% to AIDS patients and to medicines mainly prescribed for chronic diseases, with a price cap of €2.64 per prescription; medicines to hospitalized patients were provided free of charge.	After retirement: No copayment (including dependants)	NI	CBA/ BA

Additional file 1: Characteristics of the evaluated intervention

ID	Title	Place of study (Country)	Description of the drug access mechanism before the intervention	Intervention (description)	Year of the intervention implementation (4 digits)	Study design
Huh2017	Did Medicare Part D reduce mortality?	USA	Prescription Drug Discount Card: discounted prices for medicines (12–21% for brand name drugs, and 45–75% for generic drugs). Possibility of charging up to $30 for annual enrollment.	Medicare Part D (standard benefit): monthly premium of $32.20, a $250 deductible, and a coinsurance rate that begins at 25%, rises to 100% after an initial benefit limit (the “donut hole”), and then falls to 5% once the beneficiary has incurred $3600 in out-of-pocket expenditures	2006	ITS/ RMS
Kostova2017	Chronic Health Outcomes and Prescription Drug Copayments in Medicaid.	USA	No copayment	Copayment introduction	2003 and 2010	CBA/ BA
Pak2017	The impact of Medicare Part D on cognitive functioning at older ages	USA	NI	Medicare Part D (not described).	2006	ITS/ RMS
Park2017	The effect of Medicare Part D on prescription drug spending and health care use: 6 Years of follow-up, 2007-2012	USA	NI	Medicare Part D (not described)	2006	CBA/ BA
Elliott2013	Patient-centered outcomes of a value-based insurance design program for patients with diabetes	USA	30-day prescription: $10 for generic medicines, $25 for preferred brand medicines, and $50 for nonformulary brand medicines.<br>90-day prescription: $25 for generic medicines, $63 for preferred brand medicines, and $125 for nonformulary brand medicines.<br>Supplies, including syringes and glucose monitoring supplies, were covered without co-payment to an annual maximum of $7500	Co-payments for all hypoglycemic medications and supplies were eliminated	2009-2010	CBA/ BA

Additional file 1: Characteristics of the evaluated intervention

ID	Title	Place of study (Country)	Description of the drug access mechanism before the intervention	Intervention (description)	Year of the intervention implementation (4 digits)	Study design
Hanlon2013	Racial differences in antilipemic use and lipid control in high-risk older adults: Post-Medicare Part D	USA	Lower coverage of medicines insurance, possibly most of the population previous to the intervention bought their medicines out-of-pocket. However, it could had different kinds of coverage previously to the intervention.	Medicare Part D (not described): assumption of better coverage for medicines and, therefore, facilitated and better access.	2006	CBA/ BA
Nelson2014	The effect of Medicare Part D on health care utilization for non-elderly Medicare recipients with disabilities	USA	NI	Medicare Part D (not described): assumption of better coverage for medicines and, therefore, facilitated and better access to it.	2006	CBA/ BA
Ryan2014	Clinical outcomes and incremental costs from a medication adherence pilot intervention targeting low-income patients with diabetes at risk of cost-related medication nonadherence.	USA	Copayments ranged from $0 to $167.00 per prescription	Exemption from copayment	2012-2013	CBA/ BA
Lee2017	The effect of increasing the coinsurance rate on outpatient utilization of healthcare services in South Korea.	South Korea	30% of coinsurance rate on prescription medicines costs	Coinsurance rate of 50% in tertiary hospitals and 40% in general hospitals	2011	ITS/ RMS

Subtitle: USA: United States of America; NI: Not Informed; BA: before and after; ITS: interrupted time series; CBA: controlled before and after; BA: before and after; CRM: controlled repeated measure; RMS: repeated measure study.
