# Supplementary material for: Use of health outcome and health service utilization indicators as an outcome of access to medicines in Brazil: perspectives from a literature review
Source: Public Health Rev. 2019 Dec 9;40:5. doi: 10.1186/s40985-019-0115-1 (PMC6902426; doi:10.1186/s40985-019-0115-1)
Supplement: Supplementary file 2 — Evaluation of health services utilization indicators. This box presents the detailed evaluation made about each indicator of health services utilization outcome from the papers retained in this research. (PDF 63 kb) [file 40985_2019_115_MOESM2_ESM.pdf]

Additional file 2: Evaluation of health services utilization indicators

Name	Validity	Reliability	Measurability	Relevance
**Emergency**				
Use of emergency services[14]	I: depending on the conditions analyzed, its responsiveness to the pharmacological treatment and the observation time required to detect the assessment of the phenomenon	G: measure with good stability over time and in different scenarios	L: lack of a unified information system and of filling quality	G: it is related to the health condition of the user and his response to the treatment, assuming the patient's compliance to the medicine
Number of visits to the emergency department related to the DM[13]	I: depending on the conditions analyzed, its responsiveness to the pharmacological treatment and the observation time required to detect the assessment of the phenomenon. It can be more responsive to type 1 than type 2.	G: measure with good stability over time and in different scenarios	L: lack of a unified information system and of filling quality	G: it is closely related to the health condition of the user and his response to the treatment, assuming the patient's compliance to the medicine
Change in the annual number of emergency care[17]	I: depending on the conditions analyzed, its responsiveness to the pharmacological treatment and the observation time required to detect the assessment of the phenomenon.	G: measure with good stability over time and in different scenarios	I: lack of a unified information system	G: relevant outcome for public health issues
Number of visits to the emergency department[12, 16, 18, 19]	I: depending on the conditions analyzed, its responsiveness to the pharmacological treatment and the observation time required to detect the assessment of the phenomenon.	G: measure with good stability over time and in different scenarios if the same definition is used among studies	L: lack of a unified information system and of filling quality	G: it is closely related to the health condition of the user and his response to the treatment, assuming the patient's compliance to the medicine
Proportion of visits to the emergency[15]	I: in the situation, the indicator is sensitive and non-specific, since it relates to a cancer patient that, due to his health condition, is more likely to visits to the emergency.	G: measure with good stability over time and in different scenarios	L: lack of a unified information system and of filling quality	G: relevant outcome for public health issues

Name	Validity	Reliability	Measurability	Relevance
Emergency admission[22]	I: depending on the conditions analyzed, its responsiveness to the pharmacological treatment and the observation time required to detect the assessment of the phenomenon. In this study, it is a good indicator, since it is regarding inhaled medicines that quickly leads to emergency visits when it is not used regularly.	G: measure with good stability over time and in different scenarios	L: lack of a unified information system and of filling quality	G: relevant outcome for public health issues
**Hospitalization**				
Hospitalization[13, 22, 26]	I: depends on the conditions analyzed, its responsiveness to the pharmacological treatment and the observation time required to detect the assessment of the phenomenon.	G: measure with good stability over time and in different scenarios if the same definition is used among studies	L: mainly due to the filling quality of these systems	G: relevant outcome for public health issues
Number of hospitalizations[18, 19]	I: depends on the conditions analyzed, its responsiveness to the pharmacological treatment and the observation time required to detect the assessment of the phenomenon.	G: measure with good stability over time and in different scenarios if the same definition is used among studies	L: mainly due to the filling quality of these systems	G: relevant outcome for public health issues
Number of days of hospitalization[21] /Days of hospital stay [26]	I: depends on the conditions analyzed, its responsiveness to the pharmacological treatment and the observation time required to detect the assessment of the phenomenon.	G: measure with good stability over time and in different scenarios if the same definition is used among studies	L: mainly due to the filling quality of these systems	G: relevant outcome for public health issues
Changes in the annual number of hospitalization[17]	I: depending on the conditions analyzed, its responsiveness to the pharmacological treatment and the observation time required to detect the assessment of the phenomenon.	G: measure with good stability over time and in different scenarios	L: difficulty in identifying people affected with chronic and obstructive pulmonary disease	L: generic measure

Name	Validity	Reliability	Measurability	Relevance
Hospitalization use rates[20]	I: due to the characteristics of the disease and the possibility of hospitalization because of its aggravation	G: measure with good stability over time and in different scenarios	L: difficulty in identifying people affected with depression	G: relevant outcome for public health issues
Hospital utilization[24, 25]	I: depending on the conditions analyzed, its responsiveness to the pharmacological treatment and the observation time required to detect the assessment of the phenomenon.	G: measure with good stability over time and in different scenarios	L: mainly due to the filling quality of these systems	L: generic measure
Hospital admission[14, 27]	G: sensitive and specific	G: measure with good stability over time and in different scenarios	L: mainly due to the filling quality of these systems	G: relevant outcome for public health issues
Psychiatric admission[21]	G: sensitive and specific	G: measure with good stability over time and in different scenarios	L: difficulty in identifying people affected with schizophrenia	G: relevant outcome for public health issues
Risk of psychiatric admission[21]	G: sensitive and specific	G: measure with good stability over time and in different scenarios	L: difficulty in identifying people affected with schizophrenia	G: relevant outcome for public health issues
Incidence of readmission for complications related to acute myocardial infarction and death[16]	G: sensitive and specific	G: measure with good stability over time and in different scenarios	L: mainly due to the filling quality of these systems	G: relevant outcome for public health issues
Percentage of people with an inpatient admission to a hospital in 2007–09 [12]	I: depending on the conditions analyzed, its responsiveness to the pharmacological treatment and the observation time required to detect the assessment of the phenomenon.	G: measure with good stability over time and in different scenarios	L: mainly due to the filling quality of these systems	L: generic measure
**Outpatient services**				
Use of outpatient services[14, 23, 24]	I: depends on the characteristics of the intervention	I: depends on facility or difficulty of access to those services.	L: due to the filling quality of these systems	G: relevant outcome for public health issues

Name	Validity	Reliability	Measurability	Relevance
Outpatient visits[13, 21, 26]	I: depends on the characteristics of the intervention	I: depends on facility or difficulty of access to those services.	L: due to the filling quality of these systems	G: relevant outcome for public health issues
Number of outpatient visits[19, 26, 31]	I: depends on the characteristics of the intervention	I: depends on facility or difficulty of access to those services.	L: due to the filling quality of these systems	G: relevant outcome for public health issues
Number of visits to a physician[22]	I: depends on the characteristics of the intervention	I: depends on facility / difficulty of access to those services. Difficulty of comparison with international scenarios.	L: due to the filling quality of these systems	G: relevant outcome for public health issues
Number of visits to a doctor[30]	G: high responsiveness to the medicine treatment and the medicines are usually expensive	I: depends on facility or difficulty of access to those services.	L: due to the filling quality of these systems to identify specific populations	G: relevant outcome for public health issues
Number of physician office visits [18]	I: depends on the characteristics of the intervention	I: depends on facility / difficulty of access to those services. Difficulty of comparison with international scenarios.	L: due to the filling quality of these systems	G: relevant outcome for public health issues
Outpatient medical visits[16]	I: related to the characteristic of the intervention in the acquisition of cheaper medicines	I: depends on facility or difficulty of access to those services.	I: difficulty to link different databases	G: relevant outcome for public health issues
Use of ambulatory healthcare services[29]	L: generic measure	I: depends on facility or difficulty of access to those services.	G: existence of systems that permits access to the information	L: generic measure
Change in the annual number of ambulatory visits[17]	L: generic measure	I: depends on facility or difficulty of access to those services.	G: existence of systems that allows access to the information	L: generic measure
Rate of use of clinical services[20]	I: depends on the characteristics of the intervention	I: depends on facility or difficulty of access to those services.	L: due to the filling quality of these systems	G: relevant outcome for public health issues
Utilization rate of the psychiatric services[20]	I: due to the need of a prescription to buy medicine	I: depends on facility or difficulty of access to those services.	L: due to the filling quality of these systems to identify specific populations	G: relevant outcome for public health issues
Proportion of general or tertiary hospital utilization[31]	G: For the purpose of this investigation and the intervention design	I: depends on health system organization	L: due to the filling quality of these systems	G: relevant outcome for public health issues
**Total health services**				

Name	Validity	Reliability	Measurability	Relevance
Number of use of health services/100 members/month[28]	L: sensitive, because the author works with several health conditions. For this reason, it is less specific.	I: depends on facility or difficulty of access to those services.	G: existence of systems that allows access to the information	L: generic measure
**Hospital Services**				
Use of hospital health services[29]	L: sensitive, but less specific. Several factors can interfere in this measurement.	I: depends on facility or difficulty of access to those services.	G: existence of systems that allows access to the information	L: generic measure
**Diagnosis and Laboratory services**				
Use of laboratory and diagnosis services[13]	L: the indicator does not fully address to changes in the co-payment of medicine in the Brazilian scenario.	I: depends on facility or difficulty of access to those services.	L: lack of a specific registry in the information systems. In more restrictive scenario, its measure is possible.	L: only for the co-payment of medicines
**Home visits**				
Change in the annual number of home visits[17]	L: the indicator does not fully address to changes in the co-payment of medicine in the Brazilian scenario.	I: depends on the offers and regularity of this service in health systems.	L: lack of a specific registry in the information systems.	L: only for the co-payment of medicines
Other visits [26]	L: the visits included do not always will respond to lack of access to medicines, even with dialysis patients as it is the case of this study	I: depends on the offers and regularity of this service in health systems.	L: No information system to collect this data in Brazil	L: only for the co-payment of medicines

Subtitles: I: Intermediary; G: Good; L: Low
