# Supplementary material for: Use of health outcome and health service utilization indicators as an outcome of access to medicines in Brazil: perspectives from a literature review
Source: Public Health Rev. 2019 Dec 9;40:5. doi: 10.1186/s40985-019-0115-1 (PMC6902426; doi:10.1186/s40985-019-0115-1)
Supplement: Supplementary file 3 — Evaluation of health outcomes indicators; his box presents the detailed evaluation made about each indicator of health outcome from the papers retained in this research. (PDF 63 kb) [file 40985_2019_115_MOESM3_ESM.pdf]

Additional file 3: Evaluation of health outcomes indicators

Name	Validity	Reliability	Measurability	Relevance
Incidence of short-term disabilities (STD) leaves[32]	I: depends on the health condition; more sensitive for symptomatic diseases that disables the person for work	G: if the professional leaves are controlled during the study (between different moments and places)	G: overall the registries are trustworthy and stable	I: health outcome *proxy*, the focus may be on the employer rather than the user
Duration of STD leaves[32]	I: depends on the health condition; more sensitive for symptomatic diseases that disables the person for work	G: if the professional leaves are controlled during the study (between different moments and places)	G: overall the registries are trustworthy and stable	I: health outcome *proxy*, the focus may be on the employer rather than the user
Health Status[32, 37]	G: in general it represents well the need in health	G: if the instrument and conditions remain the same	L: in Brazil's case, in which data are not regularly included on a secondary basis.	G: relevant indication for public health issues
Asthma controller ratio[35]	G: a patient with good access to control treatment will present fewer episodes of urgency	G: measure with good stability over time and in different scenarios	L: due to secondary data source nonexistent; in specific scenarios, it has a good measurability potential; consumption estimate may be done from purchase data.	G: even tough it is still a *proxy*, it is closely related to the user's health condition in response to the treatment
Serum glycated hemoglobin level (HbA1c) [36, 37]	I: sensitive but not specific to medicine use, due to the multi-causality nature of diabetes control	G: measure with good stability over time and in different scenarios	L: due to secondary data source nonexistent; in specific scenarios, it has a good measurability potential	G: even tough it is still a *proxy*, it is closely related to the user's health condition in response to the treatment
Blood sugar levels[38]	I: sensitive but not specific to medicine use, due to the multi-causality nature of diabetes control	G: measure with good stability over time and in different scenarios	L: due to secondary data source nonexistent; in specific scenarios, it has a good measurability potential	G: even tough it is still a *proxy*, it is closely related to the user's health condition in response to the treatment

**Name**	**Validity**	**Reliability**	**Measurability**	**Relevance**
Change in diabetes control[39]	I: sensitive but not specific to medicine use, due to the multi-causality nature of diabetes control	G: measure with good stability over time and in different scenarios	L: due to secondary data source nonexistent; in specific scenarios, it has a good measurability potential	G: even tough it is still a *proxy*, it is closely related to the user's health condition in response to the treatment
Blood pressure [36–38]	I: sensitive but not specific to medicine use, due to the multi-causality nature of hypertension control	G: measure with good stability over time and in different scenarios	L: due to secondary data source nonexistent; in specific scenarios, it has a good measurability potential	G: even tough it is still a *proxy*, it is closely related to the user's health condition in response to the treatment
Rates of uncontrolled hypertension [41]	I: sensitive but not specific to medicine use, due to the multi-causality nature of hypertension control	G: measure with good stability over time and in different scenarios	L: due to secondary data source nonexistent; in specific scenarios, it has a good measurability potential	G: even tough it is still a *proxy*, it is closely related to the user's health condition in response to the treatment
Low-density lipoprotein cholesterol concentration[36, 37]	I: sensitive but not specific to medicine use, due to the multi-causality nature of dyslipidemia	G: measure with good stability over time and in different scenarios	L: due to secondary data source nonexistent; in specific scenarios, it has a good measurability potential	G: even tough it is still a *proxy*, it is closely related to the user's health condition in response to the treatment
Cholesterol[38]	I: sensitive but not specific to medicine use, due to the multi-causality nature of dyslipidemia	G: measure with good stability over time and in different scenarios	L: due to secondary data source nonexistent; in specific scenarios, it has a good measurability potential	G: even tough it is still a *proxy*, it is closely related to the user's health condition in response to the treatment
LDL – control[40]	I: sensitive but not specific to medicine use, due to the multi-causality nature of dyslipidemia	G: measure with good stability over time and in different scenarios	L: due to secondary data source nonexistent; in specific scenarios, it has a good measurability potential	G: even tough it is still a *proxy*, it is closely related to the user's health condition in response to the treatment
Rates of uncontrolled hypercholesterolemia[41]	I: sensitive but not specific to medicine use, due to the multi-causality nature of dyslipidemia	G: measure with good stability over time and in different scenarios	L: due to secondary data source nonexistent; in specific scenarios, it has a good measurability potential	G: even tough it is still a *proxy*, it is closely related to the user's health condition in response to the treatment

**Name**	**Validity**	**Reliability**	**Measurability**	**Relevance**
Mortality [26, 42]	I: sensitive but not specific to medicine use, several other confounding factors could influence its results	G: measure with good stability over time and in different scenarios	G: overall the registries are trustworthy and stable	G: even tough it is still a *proxy*, it is closely related to the user's health condition in response to the treatment
Mean episodic memory score[43]	I: sensitive but not specific to medicine use, due to the multi-causality nature of cognitive functioning,	G: measure with good stability over time and in different scenarios	L: due to secondary data source nonexistent; in specific scenarios, it has a good measurability potential	G: even tough it is still a *proxy*, it is closely related to the user's health condition in response to the treatment

Subtitles I: Intermediary; G: Good; L: Low
